# Jumping Stand Apparatus Reveals Rapidly Specific Age-Related Cognitive Impairments in Mouse Lemur Primates

**DOI:** 10.1371/journal.pone.0146238

**Published:** 2015-12-30

**Authors:** Jean-Luc Picq, Nicolas Villain, Charlotte Gary, Fabien Pifferi, Marc Dhenain

**Affiliations:** 1 Laboratoire de psychopathologie et de neuropsychologie, E.A. 2027, Université Paris 8, 2 rue de la liberté, 93000 St Denis, France; 2 Centre National de la Recherche Scientifique (CNRS), Université Paris-Sud, Université Paris-Saclay UMR 9199, Neurodegenerative Diseases Laboratory, F-92265 Fontenay-aux-Roses, France; 3 Commissariat à l’Energie Atomique et aux Energies Alternatives (CEA), Département des Sciences du Vivant (DSV), Institut d’Imagerie Biomédicale (I2BM), MIRCen, F-92265 Fontenay-aux-Roses, France; 4 CNRS UMR 7179, MNHN, Adaptive Mechanisms and Evolution (MECADEV), 1 Av du petit château, 91800 Brunoy, France; The Scripps Research Institute, UNITED STATES

## Abstract

The mouse lemur (*Microcebus murinus*) is a promising primate model for investigating normal and pathological cerebral aging. The locomotor behavior of this arboreal primate is characterized by jumps to and from trunks and branches. Many reports indicate insufficient adaptation of the mouse lemur to experimental devices used to evaluate its cognition, which is an impediment to the efficient use of this animal in research. In order to develop cognitive testing methods appropriate to the behavioral and biological traits of this species, we adapted the Lashley jumping stand apparatus, initially designed for rats, to the mouse lemur. We used this jumping stand apparatus to compare performances of young (n = 12) and aged (n = 8) adults in acquisition and long-term retention of visual discriminations. All mouse lemurs completed the tasks and only 25 trials, on average, were needed to master the first discrimination problem with no age-related differences. A month later, all mouse lemurs made progress for acquiring the second discrimination problem but only the young group reached immediately the criterion in the retention test of the first discrimination problem. This study shows that the jumping stand apparatus allows rapid and efficient evaluation of cognition in mouse lemurs and demonstrates that about half of the old mouse lemurs display a specific deficit in long-term retention but not in acquisition of visual discrimination.

## Introduction

As age-related cognitive impairment has become a major health problem in our societies, the need for valid animal model to investigate the biological basis of this decline and to develop efficient treatments is a crucial concern. For about two decades, there has been a growing interest in the use of a small nonhuman primate, the grey mouse lemur (*Microcebus murinus*), for studying aging and age-associated diseases [1]. Indeed, with its mouse-like body size (body length 12 cm, 60–120 g) rendering its breeding and housing cost-efficient, this nocturnal, rapidly maturing (puberty occurs at about 6–8 months) and short-lived (about a decade in captivity) primate offers a useful compromise between the practicalities and affordability of rodents and the evolutionary proximity to humans of monkeys or apes. This proximity was well illustrated by many studies that have brought out similarities of mouse lemurs and humans in the age-related changes occurring in brains, including amyloid plaque formation and neurofibrillary changes [2,3]; pathological tau metabolism [4]; neurochemical alterations [5]; neuronal loss in specific cerebral structures (e.g., the nucleus basalis of Meynert) [6]; iron accumulation [7]; and varying patterns of cerebral atrophy [8]. Interestingly, the atrophy of some brain regions such as septum, hippocampus or entorhinal cortex was only detected in a subcategory of aged mouse lemurs and was correlated with cognitive impairments, which suggests that it is related to pathological aging [9].

Concerning behavioral assessment, several studies have demonstrated that the pattern of age-related cognitive alteration in mouse lemurs [10,11] is strikingly reminiscent of that described in humans [12], namely a preserved procedural memory, a progressive and widespread decline in executive function and an impairment in declarative-like memory that is limited to a subpopulation of aged individuals. Nevertheless much work remains to be done in order to streamline the cognitive assessment of mouse lemurs. Indeed, many reports indicate insufficient adaptation of the mouse lemur to experimental devices mainly initially developed for rodents or large monkeys, leading to excessive duration of experimental trials and/or disproportionate elimination of individuals as non-responders during the training stages. For example, in a study by Ritchie et al. [13], more than 9 months of training were required for a single visual discrimination in a couple of animals. Regarding the rate of non-responders, high levels of attrition from 30 to more than 50% were often reported [14,15]. Moreover, a great inter-individual variability in the performance of control groups was frequently noted [16]. Such variability is likely to be more related to variable motivation or emotionality than to variable cognitive ability and can impede detection of subtle cognitive impairment in any experimental group. Recent progress was made to standardize cognitive testing in mouse lemurs by using an automated touchscreen-based procedure [17]. This approach has the main advantages of minimizing operator-subject interaction and facilitating cross-taxa comparisons as it can be used in a variety of species, including humans. The first publication on this apparatus reported that mouse lemurs need 24 days of training on average and more than 200 trials to reach the criterion of success assigned to a simple task of discrimination [17]. Another way to improve cognitive testing of mouse lemurs is to consider their specific behavioral and biological traits in order to design new devices enabling them to express their full cognitive potential. As mouse lemurs have an arboreal lifestyle and are powerful jumpers, developing apparatus and procedures allowing movements in the three-D space appears particularly well suited for an efficient behavioral testing of this species. In 1930, in order to study visual perception in rodents, Lashley designed a jumping stand apparatus [18]. To perform a discrimination task within this apparatus, rats had to jump from an elevated stand to one of two doors positioned in front of them. If the rat jumps to the correct door, the door swings open and the rat lands on a table behind and receives food or water reward. If it chooses wrongly, it strikes against the locked window and fall into a net slung beneath the apparatus. The jumping stand is rarely used today in rodents, because they are not prone to jumping and were forced to jump by being mildly shocked. On the contrary, mouse lemurs are jumping animals and our goal was to design a version of the jumping stand for lemurs. First, the test we designed was based on an elevated stand that can progressively be tilted downwardly causing a slippery slope pushing gently the mouse lemur to jump. Second, the target doors were replaced by target platforms that were stable for correct choices and unstable, leading to the fall of the animals, for incorrect choices. In addition, a positive reward (*i*.*e*. the possibility to attain a safe nestbox) was maintained when the animal reached the correct platform. As underscored by Sutherland and Mackintosh [19] the jumping stand technique has the great advantage that it forces animals to look toward the stimuli before responding (usually for some time as they normally hesitate before leaping the gap) and it gives immediate positive or negative reinforcement after a correct or an incorrect jump. The main goal of the present study was to determine whether the jumping stand apparatus adapted to mouse lemurs allows obtaining fast and efficient learning and could be used as a reliable tool for testing cognition in this species.

The second goal of our study was to test whether the jumping stand apparatus can evaluate age-related cognitive alterations in mouse lemurs. The jumping stand apparatus can assess different cognitive abilities through a large panel of tasks such as simple discriminations, delayed matching (or nonmatching) to samples or shifting tasks. Acquisition and retention of simple discrimination learning task are differentially sensitive to medial temporal lobe lesions [20,21,22] and as temporal lesions are altered in a subcategory of old mouse lemurs [9], impairment was expected in retention but not in acquisition of discrimination problems. We thus designed discrimination and retention tasks based on the jumping stand apparatus to evaluate medial temporal lobe-dependent and non-medial temporal lobe-dependent cognitive performance in young and old mouse lemurs.

## Materials and Methods

### 2.1. Ethics statement

The study was non-invasive and carried out in accordance with the European Communities Council Directive (2010/63/UE). The research was conducted under the approval of the CETEA-CEA DSV IdF ethic committee under the authorization number 12–089. In accordance with the recommendations of the Weatherall report, “The use of non-human primates in research”, special attention was paid to the welfare of the animals. Neither nociceptive stimuli nor food deprivation were used during this work.

### 2.2. Animals

Twenty male grey mouse lemurs (*Microcebus murinus*) were evaluated. The young adult group consisted of 12 animals ranging from 3 to 4.2 years (mean age = 3.3 years) and the aged adult group consisted of 8 animals ranging from 7 to 10 years (mean age = 7.5 years). These age categories were consistent with age classification in previous studies and were based on survival data of the breeding colony which has a mean and a maximum lifespan of 56 and 120 months, respectively [1]. The animals were born and reared in the Brunoy colony (MNHN, France, licence approval N° A91.114.1). They had no previous experience with cognitive testing nor with any drug trial or experimental surgeries. The mouse lemurs, solitary foragers in the wild, were housed in individual cages to reduce stress due to capture and transport to the separate experimental room. The cages were enriched with tree branches and wooden nests (nestbox) and were kept at standard temperature (24–26°C) and relative humidity (55%). The mouse lemurs were tested during the summer-like long day length (14:10 hours light-darkness) that corresponds to the active phase of the animals. Animals were raised on fresh fruits and a laboratory daily-made mixture of cereals, milk and eggs. Water and food were given *ad libitum*. The eyes of the mouse lemurs were examined by a veterinary ophthalmologist and no anomalies were detected that would affect visual acuity.

### 2.3. Apparatus

**Fig 1:**
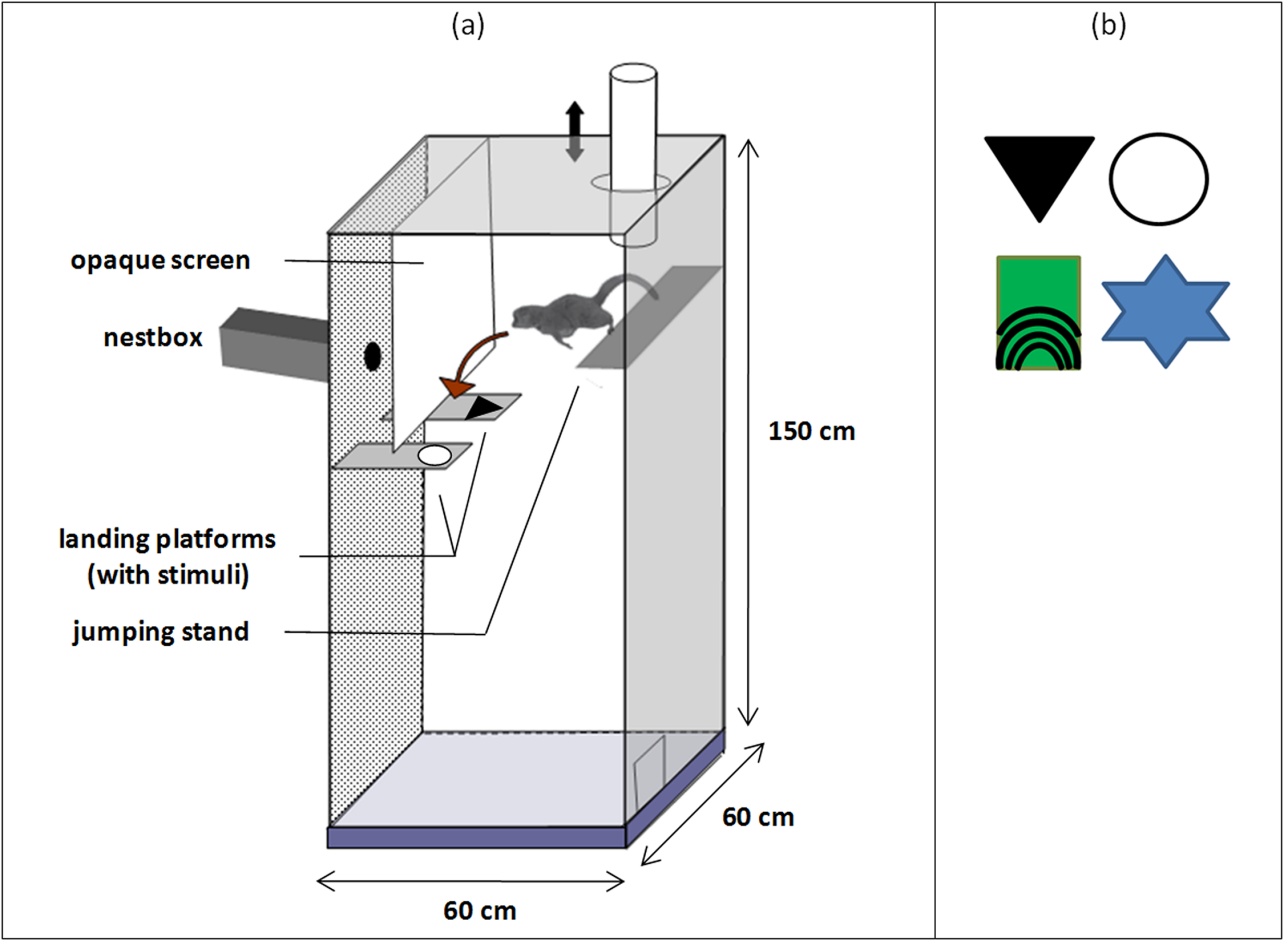
Jumping stand apparatus for mouse lemurs (a) and pairs of stimuli used for the two discrimination problems (b).

Experiments were performed in a separate testing room containing the apparatus. The apparatus (see Fig 1) was a big vertical cage (height = 150 cm) made of plywood walls except the front panel that was a one-way mirror to allow observation. It was illuminated by 50-W bulb attached to the center of the cage ceiling. The mouse lemur was placed inside the cage on the center of an elevated plastic starting platform (the jumping stand) through an open-ended dark cylinder that opened in the roof of the cage. After this cylinder was gently lifted, the mouse lemur was required to jump from the elevated stand onto one of two landing platforms (15 cm x 30 cm) across a 35-cm gap. If it did not jump within one minute the starting stand was progressively tilted downwardly causing a slippery slope pushing the mouse lemur to jump. If it jumped onto the correct platform, it could pass under an opaque Plexiglass screen to access its nestbox placed behind the wall of the cage. This opaque screen prevented mouse lemurs from jumping directly to the opening of the nestbox. If it jumped onto the incorrect platform, the platform swung down and the mouse lemur fell into the bottom of the cage on a wide soft pillow to avoid any risk of injury. A small door at the base of the right wall of the cage allowed to take back the mouse lemur and to put it back on the starting platform for another trial. The correct and the incorrect platforms were distinguished by visual stimuli placed on them. The training pairs of stimuli were randomly drawn from a pool of 20 pairs. Within each pair, stimuli were chosen to be easily discriminable as the goal was to test cognitive and not visual capacities. Accordingly the two stimuli of the same pair differed both in shape, texture, brightness or pattern. Practically, they were made of different materials (cardboard, plastic, polystyrene, fabric…).

No food reinforcement was used. The reward for positive choices consisted in allowing the mouse lemur to reach its nestbox and then to be "home safe" for some time. This reward is particularly efficient in mouse lemurs as they are very keen to find their nestbox, due to their habit in their natural environment to make use of tree holes as vital means to evade predation, ensure thermoregulation, and park infants during food foraging [23].

### 2.4. Testing procedure

#### 2.4.1. Overview of the performed tasks

**Fig 2:**
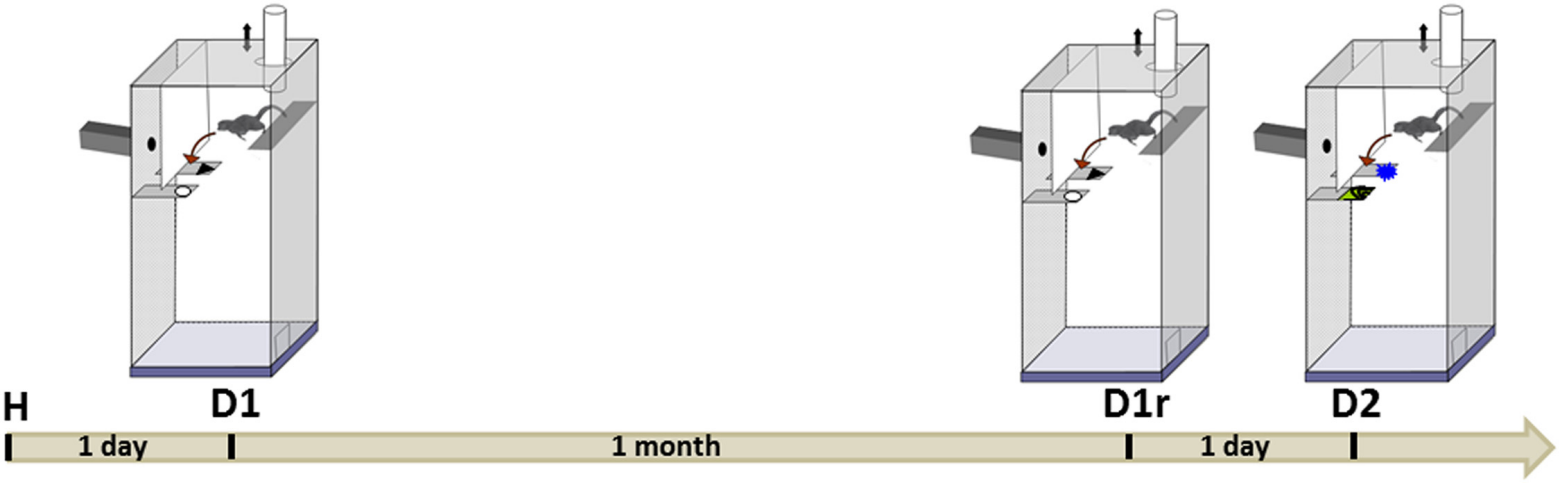
Timeline of tasks. H: habituation, D1: first discrimination problem, D1r: retention of the first discrimination problem, D2: second discrimination problem.

Two discrimination problems (D1 and D2) and a long term-memory test (retention phase) of the first discrimination problem (D1r) were used (see Fig 2). During the first discrimination problem (D1), the mouse lemurs had to discriminate two visual stimuli. The mouse lemurs were given each day a session of a maximum of 25 trials. Testing continued until the mouse lemurs reached a criterion of eight correct choices for ten consecutive trials. The long term-memory test (D1r) was performed one month after the first discrimination problem. The following day a new discrimination problem (D2) was performed. For each test, the score was the number of errors (wrong choices leading to a fall) before reaching the criterion. One day before the first discrimination test, each mouse lemur was given a habituation session to learn the task.

#### 2.4.2. Habituation

The habituation session was composed of seven trials. For the first four trials, only one fixed central landing platform was attached just below the nestbox opening. On trial 1, a cylindrical rod connected the jumping stand to the landing platform so that no jump was required to attain the nestbox whose opening was visible from the starting stand. On trial 2, the rod was removed so that the mouse lemur had to jump onto the central landing platform to access its nestbox. On trials 3 and 4, an opaque vertical screen was added above the middle of the landing platform masking the nestbox opening. The mouse lemur had to jump onto the central landing platform then to walk under the screen to access its nestbox. For the last three trials, the fixed landing platform was placed alternatively to the left or to the right of the nestbox opening which was masked by the opaque screen.

#### 2.4.3. Discrimination tests

During the discrimination problems (D1, D1r, and D2), mouse lemurs were given each day a session of a maximum of 25 trials. On each trial, the mouse lemur faced two landing platforms. It had to choose the positive landing platform which gave access to a 2-min rest in its nestbox and to avoid the negative platform which resulted in the fall of the platform leading to the drop of the animal in the bottom of the cage. After a fall, the mouse lemur was left in the bottom for 20 seconds before being taking back for another trial. The discrimination was based on visual stimuli that differed in shape, texture, brightness or pattern. These stimuli (diameter about 15 cm) were interchangeably attached to the front of the landing platforms. The location of the stimuli on the right or left landing platforms was randomized with the restriction that the positive stimulus was not located on the same platform for more than three consecutive trials. Two different pairs of stimuli were selected for the two discrimination tests. One pair consisted of a black plastic triangle versus a white cardboard circle and the other pair consisted of a green rubber rectangle with black concentric lines versus a blue star-shaped rigid paper (Fig 1b). The order of the two discrimination problems was reversed for half the animals.

### 2.5. Data analysis

All values are expressed as mean ± standard error of the mean (SEM). Performances between tasks (acquisition, retention, new acquisition) were compared in each age-group with paired Student’s t-test analyses and performances between young and old animals on each task were compared with unpaired Student’s t-test analyses. The level of statistical significance was p < 0.05.

## Results

The general performances of young and old mouse lemurs in the D1, D1r and D2 tasks are presented in Table 1. All tested animals succeeded in acquiring the first visual discrimination (D1) in one or two sessions. The mouse lemurs needed from 13 to 41 trials to reach the criterion (median = 26 or 24 for young animals or aged animals, respectively). Thus the first discrimination could be learnt in only two days. Evaluation of the animals for the long term-memory test (D1r) or the new discrimination problem (D2) was also performed very rapidly, *i*.*e*. within one day (Table 1).

**Table 1:** Mean ± SEM number of days and trials to reach the success criterion in the jumping stand apparatus. The figures in brackets indicate the score range. Attrition rate was null for each task.

	D1		D1r		D2	
	Days	Trials	Days	Trials	Days	Trials
**Youngs**	2 (1/2)	26±2 (13/41)	1 (1/1)	9±0 (8/10)	1 (1/1)	15±1 (9/23)
**Olds**	2 (1/2)	24 (14/40)	1 (1/1)	12±1 (8/18)	1 (1/1)	14±1 (10/22)

**Fig 3:**
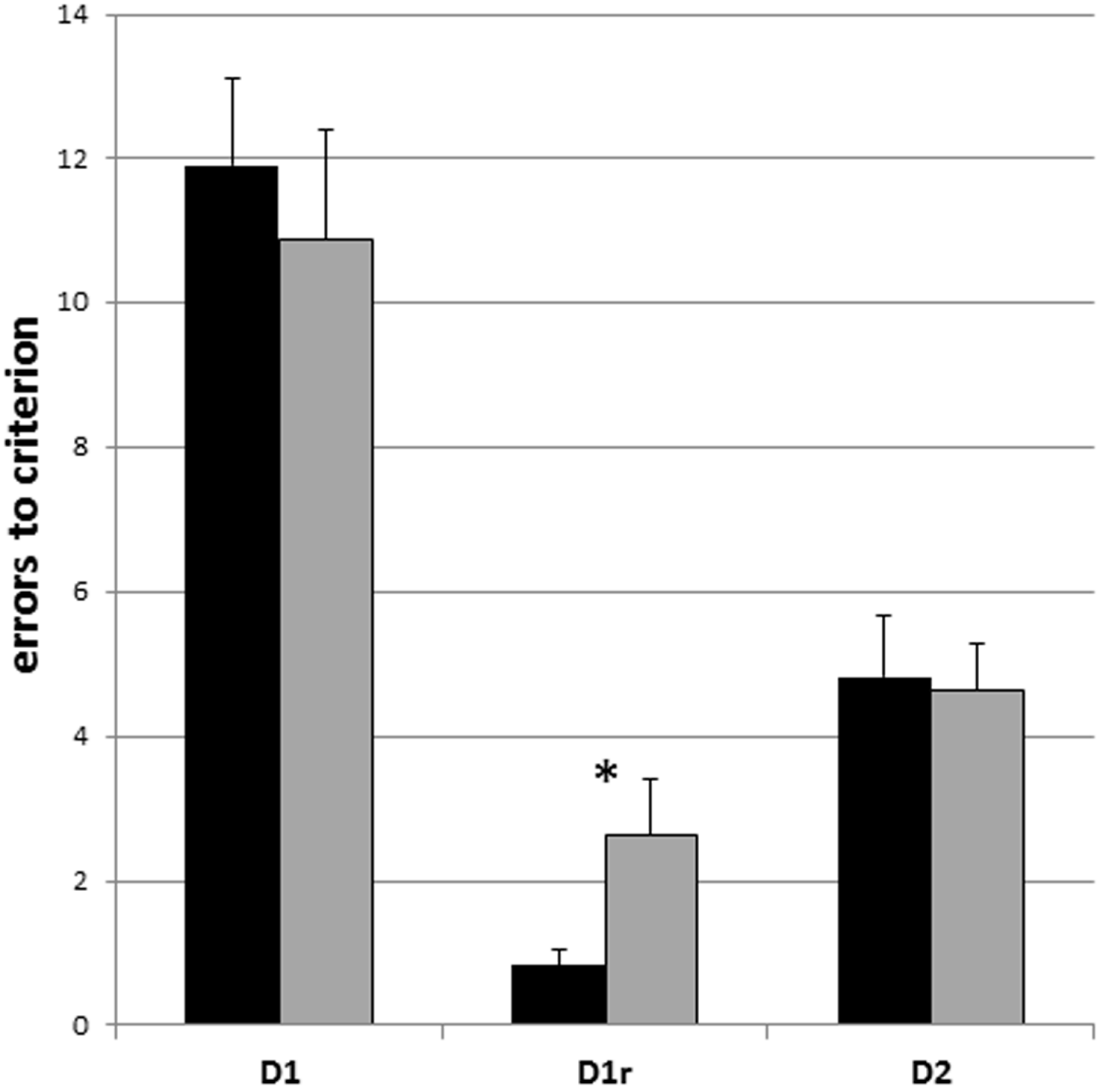
Mean scores on the first discrimination problem (D1), retention of the first discrimination problem (D1r) and second discrimination problem (D2) in young (black) and older (grey) adult animals. Errors bars depict SEM. * indicates a significant difference.

In the first visual discrimination (D1) the mean number of errors to criterion was 11.9 ±1.2 for the young group and 10.9 ±1.5 for the aged group (see Fig 3 and S1 Table). No group differences were observed between young and old individuals (t = 0.53, p > 0.05; Fig 3). On the retention phase of the first discrimination problem (D1r), both young and old animals succeeded faster than in the learning of the discrimination (D1) (t = 9.68 and 6.25, respectively, p < 0.001). All young mouse lemurs immediately attained at least a 80% correct response level (criterion level) in the first ten trials (errors ≤ 2, mean = 0.8 ±0.2). The older animals made more errors before reaching criterion (mean = 2.6 ±0.8) than the young ones (t = 26, p = 0.018) and only half of the aged mouse lemurs succeeded in reaching the criterion in the first ten trials (Fig 3). No differences between young and aged mouse lemurs were seen on the second discrimination problem (D2, t = 0.17, p > 0.05). The performances on D2 were significantly better than those recorded on D1 for both the young and old individuals (t = 6.72 and 4.64, respectively, p < 0.01; Fig 3). The performances on D1r were significantly better than those recorded on D2 for the young individuals (t = 5.07, p < 0.01) but not for the aged individuals (t = 2.25, p > 0.05).

## Discussion

The main goal of our study was to determine whether the jumping stand technique is an efficient way to test the cognitive capacities of mouse lemurs. Our findings demonstrate that naive mouse lemurs are able to master a visual discrimination task in a very small number of trials (13 to 41) after a short habituation session of only seven trials. Even though the criterion to be reached was not the same, young adult mouse lemurs needed far fewer trials (median = 26) to acquire a pairwise discrimination in the jumping stand task than in earlier protocols involving odor discrimination [24], visual discrimination using the touchscreen procedure [17] or visual discrimination of illuminated vs. dark corridors [11], which required about 500, 200 or 80 trials, respectively. The cognitive demand being identical in all these discrimination tasks, the excellent performance of mouse lemurs in the jumping stand apparatus was likely based on sensory, motor, attentional and motivational parameters that make this procedure particularly appropriate to their biology. Indeed, in the jumping stand procedure, the visual stimuli were easily detectable and discriminable by mouse lemurs. Also attention was quickly focused on these stimuli as the mouse lemurs were strongly motivated to escape from the well-lighted and very exposed slippery stand and no other target was available. Moreover, the required jump response is a central part of the spontaneous locomotor behavior of this arboreal species. Finally, choices were instantly either rewarded (access to the nestbox) or punished (fall) making the conditioning procedure more powerful byCombining positive and negative reinforcements across trials. Moreover rewards and punishments are ecologically significant for mouse lemurs in this protocol since falling from an unstable support is a natural risk for an arboreal jumping species. In accordance with the high performances of mouse lemurs in the jumping stand, which suggests that this test procedure is relatively straightforward for this species, the attrition rate recorded in the present study over the whole experiment was zero. Given the relatively limited number of mouse lemurs (in comparison to rats or mice) available for laboratory studies, it is important that as few animals as possible have to be excluded as non-responders before completing a cognitive test battery. Another advantage of the jumping stand apparatus is that simple cognitive tasks such as visual discrimination tasks carried out in this device did not result in excessive inter-individual variability in young adult control group. Indeed most young adult mouse lemurs (11 out of 12) needed from 20 to 40 trials to reach criterion. The variability among young individuals in the present study was even far more limited in the retention test (D1r), since all mouse lemurs needed only from 8 to 10 trials to reach criterion. Thus discrimination tasks in the jumping stand device seem sufficiently sensitive to detect mild cognitive deficit in aged subjects (or in any other experimental group) and to reach statistical significance with a relatively low number of animals. Another interest of the jumping stand apparatus is that it will be easy in further studies to test separately each dimension (shape, size, texture, color, pattern) of the visual stimulus and to determine which dimension is most relevant for the mouse lemur and whether one dimension can induce age-related impairment. Moreover intra-dimensional and extra-dimensional shift tasks, which are known to be especially vulnerable to aging in primates [25,26,27], could easily be carried out.

In our study, the older mouse lemurs learned the first visual discrimination problem with a level of performance similar to that of the young ones. All aged animals performed inside the range score of the younger group. Thus the motor requirement of the jumping stand procedure was moderate enough not to impede cognitive capacity expression in aged individuals. The lack of age-related cognitive impairment in discrimination learning is consistent with previous studies in mouse lemurs [9,11,24] and has been frequently reported in rodents [28] and monkeys [29,30,31]. To the extent that such tasks can be solved on the basis of acquisition of attentional and motor biases towards individual stimuli, there are thought to mainly involve procedural memory, a form of memory well preserved in the human aging process [32,33]. Nevertheless a recent study using a touchscreen device found that aged mouse lemurs are impaired in the acquisition of a visual discrimination between two images [17]. Strong differences between the jumping stand and the touchscreen procedures concerning many parameters such as the required motor response, the type of reinforcers used and the nature of visual stimuli to discriminate can suffice to explain the contrasting observations.

Both young and aged mouse lemurs learned the second discrimination problem (D2) much more quickly than the first one and the two age groups showed a similar level of performance. This improvement in the second problem likely relies on memory of the general rules of the discrimination task (transfer effect) in the jumping stand apparatus, supporting rapid solutions of new problems of the same class. However, although both young and old animals performed better on the retention task (D1r) than on the first discrimination (D1), the performance of the young group was better than that of the old animals in the retention task (D1r). Young animals showed a perfect recall of the specific stimulus discrimination problem a month after criterion was met. This higher level of accuracy in the retention test of the first discrimination problem cannot be ascribed to rapid acquisition of a learning set in young animals because their performance decreased in the following second discrimination problem when compared to the retention problem. This suggests that long term retention of the general rules of discrimination problems and long term retention of a specific discrimination problem have to be differentiated. Our data show that, while all the young animals were able to reach the criterion in the first ten trials of the retention test, only half of the aged animals were able to do so. The old mouse lemurs with worse scores performed as well as the young adults in both the first and the second discrimination problems. These results suggest that some aged mouse lemurs can display deficit in long term retention of a specific stimulus discrimination problem and be perfectly capable to master discrimination learning and to acquire a learning set by retaining the general rules of these tasks. This acquisition-retention distinction in discrimination tasks has been supported by experiments that demonstrated that rats with entorhinal/hippocampal lesions acquired successive discrimination problems in the same amount of time as did controls but showed marked deficits in the retention phase of these discrimination problems after long intervals of time [22]. This selective retention impairment suggests that medial temporal lobe structures are important for retaining specific visual discriminations over long delay intervals but neither for mastering a discrimination task nor for improving performance over successive discrimination problems [21,34]. This gradual gain in performance across problems is probably partly due to progressive acquisition of the procedural aspects of testing with experience and more dependent on corticostriatal circuits [35]. The present findings are consistent with previous data from our team that revealed age-related hippocampal and entorhinal atrophy in a subpopulation of aged mouse lemurs and a relationship between that atrophy and poor spatial memory performance in the circular platform test [9]. Thus we hypothesize that poor performance in the retention test of specific discrimination problems after long delays is sensitive to entorhinal/hippocampal dysfunction. The procedure described in the present study might detect aged mouse lemurs affected by pathological aging processes involving these areas. Further magnetic resonance imaging and histological evaluations of the brain of mouse lemurs tested in the jumping stand will help to explore this hypothesis.

In conclusion, the jumping stand apparatus appears to be a powerful tool for testing cognition in mouse lemurs and for exploring age-related impairment. Its efficiency can be explained because the apparatus is designed for the arboreal lifestyle of the lemurs as well as for their sensorial and behavioral skills. We suggest that it can also be adapted to evaluate cognitive abilities of other arboreal animals.

## Supporting Information

S1 Table Individual performances of the mouse lemurs. (DOCX)
