# Supplementary material for: Jumping Stand Apparatus Reveals Rapidly Specific Age-Related Cognitive Impairments in Mouse Lemur Primates
Source: PLoS One. 2015 Dec 30;10(12):e0146238. doi: 10.1371/journal.pone.0146238 (PMC4696676; doi:10.1371/journal.pone.0146238)
Supplement: S1 Table — (DOCX) [file pone.0146238.s001.docx]

Table S1. Individual performances of the mouse lemurs.
